# Physiological and Proteomic Approaches to Address the Active Role of *Botrytis cinerea* Inoculation in Tomato Postharvest Ripening

**DOI:** 10.3390/microorganisms7120681

**Published:** 2019-12-11

**Authors:** Nikolaos Tzortzakis

**Affiliations:** Department of Agricultural Sciences, Biotechnology and Food Science, Cyprus University of Technology, 3603 Limassol, Cyprus; nikolaos.tzortzakis@cut.ac.cy

**Keywords:** tomato fruit, gray mould, storage, proteomics, quality

## Abstract

*Botrytis cinerea* is an unbearable postharvest threat with significant economic impacts. Necrotrophic *B. cinerea* can readily infect ripe fruit resulting in the rapid progression of symptoms of the disease. To unravel the mechanism by which tomato fruit opposes pathogen attack, we investigated the changes in quality-related attributes as a direct response (DR) or systemic response (SR) of infected tomatoes to the *B. cinerea.* Additionally, the SR of protein yield and composition were studied in fruit stored at 11 °C/90% relative humidity (RH) for one week. Fungal infection accelerated ripening with increased ethylene and respiration rates. Fruit softening, ascorbic acid and β-carotene increase were associated with DR but not with the SR of the pathogen. Pathogen infection increased lipid peroxidation, causing the production of hydrogen peroxide and oxidative stress, as fruit activated both enzymatic and non-enzymatic mechanisms to trigger stress. *B. cinerea* increased up to 6.6% the protein yield and downregulated at least 39 proteins. Proteins involved in fruit ripening, such as an ethylene biosynthetic enzyme, were increased in wound-inoculated fruit. Moreover, antioxidant proteins, such as ascorbate peroxidase-APX1 and superoxide dismutase-SOD, increased in infected tomatoes, as these proteins are involved in reactive oxygen species detoxification. Constitutively-expressed proteins tended to be either increased (chaperonin and malate dehydrogenase) or remained unaffected (dehydrin) by pathogen inoculation. Protein levels involved in the metabolism of carbohydrate, the pentose phosphate pathway, terpenoid and flavonoid biosynthesis were differently affected during the treatments. By enabling a better understanding of the fungal direct or systemic response on fruit quality and ripening through biochemical and proteome studies, we may improve the plant–pathogen interaction and complexity.

## 1. Introduction

*Botrytis cinerea* (Pers.) [tel. *Botryotinia fuckeliana*] is considered the second most important fungal pathogen of plants after *Penicillium expansum*, with preharvest and postharvest rotting infections [1,2]. It is a generalist necrotrophic pathogen causing major postharvest losses with significant economic impacts on various crops, as rotting can be on fruit, vegetables, flowers and leafy ornamentals, and serves as a model species for plant–necrotroph interactions [3]. *B. cinerea* causes grey mould disease in many fresh commodities from the Solanaceae (tomatoes, peppers and eggplants), Curcubitaceae (melons, cucumbers, squash and pumpkin) and other economically sound produce [4]. The levels of postharvest losses of fresh commodities caused by microorganism attack worldwide range from 10% to 30% of the total crop yield, with significantly higher losses in developing countries [5]. Tomato is regarded as a major horticultural crop providing a considerable nutritive value of vitamins (C, B, A), minerals (calcium, potassium, magnesium, iron) and antioxidant capacity [6,7]. Tomato preferred storage conditions are 10–12 °C for reducing the metabolic processes related to ripening/maturation, while lower (i.e., 7 °C) temperatures may cause chilling injuries and physiological disorders and shorten the postharvest life of the commodity. Chilling injury causes the release of metabolites, such as amino acids and sugar, and minerals from cells, and together with the degradation of cell structure, challenge the pathogen infection. These detrimental changes reduce quality and consumer acceptability leading to substantial economic loss.

Optimal temperatures for *B. cinerea* growth is 18–23 °C, however, some growth will occur even at cool storage temperature (usually 0–5 °C), when fruit resistance is decreased by cell degradation through ripening and maturation of the fresh produce [8]. Despite several postharvest preservation means examined in tomatoes, such as ozone [9,10,11], essential oils [12,13,14], acetic acid [15], chitosan, UV-C [16] and heat treatment [17], a chilled storage temperature is a necessity for postharvest preservation of fresh produce. Application of synthetic chemical compounds, chilled storage, and modified atmosphere storage techniques are, therefore, the primary means of controlling postharvest decay, including gray mould, of fruit vegetables [5].

During active infection, *B. cinerea* secrets cell wall degrading enzymes and yields toxins [3]. Additionally, susceptibility to *B. cinerea* changes during development and age, and this is related to several metabolic processes, including increasing ethylene and respiration rates (especially in climacteric produce) [7,18], enzyme degradation and cell-wall loosening [19], soluble sugar accumulation, pH changes [20], cuticular changes [21], decline of preformed and inducible antifungal compounds and secondary metabolites [20], to name a few. Most of these changes are regulated by hormone signals, including ethylene, abscisic acid, jasmonic acid and salicylic acid, which increase during fruit ripening [18,22]. Noticeably, similar phytohormones are regulated in the host defense response to pathogens [23,24]. These changes are sensed by the pathogen and switch to the necrotrophic lifestyle and cause decay [20]. 

Quality attributes affect nutritional value, maintenance during storage, and safety for consumers. Moreover, unripe tomatoes [25] and strawberries [26] were more resistant to *B. cinerea* infection compared to the ripe fruit. Petrasch et al. [26] reported that sugar accumulation in ripe strawberries could serve as nutrients for *B. cinerea*, however, Blanco-Ulate et al. [27] suggested that fungi susceptibility is not necessarily related to high levels of sugars in tomato mutants, but sugars could still influence the pathogen susceptibility as they can serve as ripening initiation signals. The plant–pathogen interaction system is complicated and still under exploration, with plant hormones biosynthesis and signaling changes taking place with pathogen attack [26]. Blanco-Ulate et al. [23] reported that *B. cinerea* could initiate ethylene production in unripe tomato fruit and thereby stimulate early ripening. Of note, the pathogen and plant interaction on the quality of fresh produce is not well examined, and limited proteomic data on this interaction is available. Mainly proteomic studies were focused on plant proteomes, and fewer are catalogued in infected tissues by a pathogen [28]. Even more limited information is available on proteome changes in the healthy part of an infected fruit [29]. Therefore, the proteome profile of the powdery mildew (*Erisphye pisi*) infection on pea [30], *Pseudomonas syringae* infection of *Arabidopsis* [31], *Xanthomonas campestris* on *Brassica oleracea* [32] revealed the identification of less than 100 plant-related proteins. Thus, most protein identifications were related to the pathogen itself. Moreover, *B. cinerea* proteome had been identified from cultures of fungal (mycelium) pathogens grown on synthetic media [33,34], resulting in more than 300 identified proteins. Proteomic analysis of *B. cinerea* secretome (extracts of ripped tomato and strawberry and *Arabidopsis* leaf extract) has also been studied [35]. 

Proteomics approaches were used in tomatoes (*Solanum lycopersicum* Mill.) to examine various aspects including responses to pathogens [36,37], viruses [38,39], abiotic-stresses [40,41], interaction of abiotic (ozone) and biotic (pathogen) stress [29] as well as metabolism and development [42,43,44]. Casado-Vela et al. [39] reported that when tomato tissue infected with tobacco mosaic virus was sampled and analysed for proteome changes, the expression of several pathogenesis-related (PR) proteins and antioxidant enzymes were identified as a response resistance of tomato to viral infection. Previous proteome studies heat-stressed or blossom-end rot tomato fruit revealed induction of antioxidant metabolism (ascorbate–glutathione cycle) and the pentose phosphate pathway indicating that these two biochemical pathways could be related with the increased scavenging of reactive oxygen species (ROS) in tomato fruit [45,46,47]. 

In the present study, the impacts of *Botrytis cinerea* infection on the qualitative and antioxidant traits for tomato fruit were investigated. Quality attributes were determined in both fruit sides, the fungi infected part (direct response-DR) and the opposite site of non-pathogenic symptoms (systemic response-SR). Unlike previous studies of proteins secretion by *B. cinerea,* including either the identified fungal proteins produced in cultures or the proteins reflected on both organisms simultaneously, this is the first analysis of the protein changes which are related to the healthy part of the infected fruit and the plant–fungal pathogen interaction. In that direction, a metadata analysis was observed following our previous study when examined the systemic response of the effects of ozone on healthy and wound-inoculated fruit with *B. cinerea* [29]. The systemic response of *B. cinerea* inoculation on tomato fruit has not been targeted previously, and this is addressed with the present analysis. Therefore, we examined the responses of *B. cinerea* infected tomatoes a) on the fruit quality at the infected and non-infected parts of the fruit b) and tomato proteins changes in the non-infected part of the fruit as a systemic response to the *B. cinerea* infection. 

## 2. Materials and Methods

### 2.1. Fungal Culture

*Botrytis cinerea* isolated from tomato (strain number: 169558) was supplied by CABI (CABI Bioscience UK Centre, Bakeham Lane, Egham, England). Fungal culture, grown in potato dextrose agar (PDA), was stored at 4 °C for long-term use.

### 2.2. Plant Material and Experimental Design 

Freshly harvested red ripe (mean weight of 87.97 g, firmness of 26.6 N, total soluble sugars content of 228.32 μmol/g fresh weight) tomato fruit (*S. lycopersicum* Mill. cv. Carousel) were selected based on uniform size (49–58 mm), appearance, and absence of physical defects and used immediately in the different experiments. Fruit were disinfected in chlorine (0.05% v/v) solution before use, to avoid any microbial load.

Fruit inoculation was performed as described in Tzortzakis et al. [10]. Briefly, tomatoes were divided into four replicate batches (each batch containing five fruit), and one wound (3 mm diameter and 2–3 mm deep) was made on every fruit using a sterile scalpel. *B. cinerea* spore suspension of 7-day-old colonies was collected, quantified with the aid of a haemocytometer and 5 μL of 2 × 10^4^ spores was inoculated into the wounds made in each fruit. Fruit remained for 3 h at room temperature to ensure successive spores establishment. Four replicates batches (each batch containing five fruit) of non-inoculated fruit were considered as control treatments. Each batch of tomatoes was placed in individual 5 L polystyrene containers with snap-on lids and stored in chilled conditions for 1 week at 11 °C and 90% relative humidity (RH) (Figure S1). Filter paper was moistened with distilled water and placed in a small beaker and was subsequently placed into each container, remoistened every second day, maintaining high RH during the storage period, as described in Chrysargyris et al. [48]. Containers lids were opened every 48 h and aerated to prevent air composition abnormalities. 

### 2.3. Quality and Ripening-Related Attributes

The rates of respiration and ethylene production were determined every second day throughout the storage period, as described previously [48]. Briefly, each fruit was placed in a 1 L glass container, sealed with a rubber stopper and incubated for 1 h, at room temperature. Carbon dioxide (CO_2_) and ethylene production were measured by a Dual gas analyser (GCS 250 Analyzer, International Control Analyser Ltd., Kent, UK) and ethylene analyser (ICA 56 Analyser, International Control Analyser Ltd., Kent, UK), respectively. Fruit weight and volume were recorded to calculate the relevant rates. Results were expressed as millilitres of CO_2_/kg/h and microlitres of ethylene/kg/h, respectively. Eight replicates were used for control and wound-inoculated fruit with *B. cinerea* for each sampling date.

Following 1 week of storage, fruit tissue of four biological replicates (pool of two tomatoes/replicate) for inoculated and control fruit were measured/sampled. In wounded fruit, plant tissue was measured/sampled from the infected side of the fruit (0.5 cm away from the margins of the lesion area) and a second plant tissue measured/sampled on the opposite side of the wound (i.e., ‘healthy tissue’) as our aim was to check the direct (DR) and the systemic (SR) response of fruit to wounding/pathogen. 

Fruit weight was recorded initially and after 1 week of storage, and weight loss was calculated for each fruit per treatment. Fruit firmness was measured at 1 point on the shoulder of each tomato for the infected area (0.5 cm away from the margins of the lesion area) and opposite the area of the fungal lesion by a texturometer FT 011 (TR Scientific Instruments, Forli, Italy) and results expressed in Newtons (N). Colour was measured using the Hunter Lab System and a Minolta colourimeter model CR400 (Konica Minolta, Osaka, Japan). Following the recording at 2 points for the direct and systemic areas of each tomato for the individual L*, a*, and b* parameters, chroma value (C) was calculated by the following equations C = (a^*^^2^ + b^*^^2^)^1/2^ as described previously [49]. 

Tomato juice was obtained from 2 pooled tomatoes for each replication (*n* = 8), and total soluble solids (TSS, expressed in percent) measured with a temperature-compensated digital refractometer (model Atago PR-101, Atago Co. Ltd., Tokyo, Japan) at 20 °C. The titratable acidity (TA) was measured via potentiometric titration (Mettler Toledo DL22, Columbus, Ohio, USA), and results were expressed as percentage of citric acid. Ascorbic acid (AA) was determined by the 2,6-Dichloroindophenol titrimetric method as described previously [49] and results expressed as milligram of AA/g of fresh weight (Fw). Carotenoids (lycopene and *β*-carotene) were measured based on Nagata and Yamashita [50] following modification [48]. In brief, 1 g of blended tomatoes was placed in 50 mL falcons and 16 mL of acetone:hexane 4:6 (v:v) was added to each sample, the samples were shaken vigorously, and the two phases were separated automatically. An aliquot was taken from the upper solution for measurement of optical density at 663, 645, 505 and 453 nm in a spectrophotometer, using a reference acetone:hexane (4:6) ratio. Lycopene and *β*-carotene contents were calculated and expressed in nanomole/gram of fresh weight [50].

Methanolic extracts of tomato fruit were used to determine the content of total phenols, as previously described [51]. In brief, tomato tissue (0.5 g) was milled (for 60 s) with 10 mL methanol (50% v/v), and extraction was assisted with ultrasound for 30 min. The samples were centrifuged for 15 min at 4000 *g* at 4 °C (Sigma 3–18 K, Sigma Laboratory Centrifuge, Osterode, Germany). Total phenols content was determined using the Folin–Ciocalteu method at 755 nm, according to Tzortzakis et al. [51], and results were expressed as milligram of gallic acid equivalents (GAE) per gram of fresh weight. Antioxidant capacity was determined by a ferric-reducing antioxidant power (FRAP), 2,2′-azino-bis(3-ethylbenzothiazoline-6-sulphonic acid (ABTS) and 2,2-diphenyl-1-picrylhydrazyl (DPPH) radical-scavenging activity assays as described previously [49,52]. FRAP radical scavenging activity of the plant extracts was measured at 593 nm. ABTS scavenging capacity was measured at 734 nm. DPPH radical scavenging activity of the plant extracts was measured at 517 nm from the bleaching of the purple-coloured 0.3 mM solution of DPPH. The results were expressed in milligram Trolox per gram of fresh weight.

The fruit damage index was observed by hydrogen peroxide (H_2_O_2_) and lipid peroxidation through the malondialdeyde (MDA) content, as described previously [53]. In brief, tomato tissue (0.2 g) was homogenised in ice-cold 0.1% trichloroacetic acid (TCA) and centrifuged at 15,000 *g* for 15 min. An aliquot of the supernatant was mixed with 0.5 mL of 10 mM potassium–phosphate buffer (pH = 7.0) and 1 mL of 1 M potassium iodide. The H_2_O_2_ concentration was evaluated using standards prepared from dilutions of H_2_O_2_. The absorbance was measured at 390 nm, and results were expressed as nanomole H_2_O_2_ per gram fresh weight. For MDA determination, the reaction mixture of 0.5 mL of the above extract and 1.5 mL of 0.5% thioarbituric acid (TBA) in 20% TCA was incubated at 95 °C for 25 min and cooled. Absorbance was measured at 532 nm and corrected at 600 nm. The amount of MDA was determined using the extinction coefficient of 155/mM/cm. Results were expressed as nanomole of MDA per gram fresh weight. Enzymes antioxidant activity (catalase-CAT, ascorbate peroxidase-APX, peroxidase-POD and superoxide dismutase-SOD) was determined according to Chrysargyris et al. [53]. Briefly, homogenised fresh tomato samples were extracted with ice-cold extraction buffer [1 mM ethylenediaminetetraacetic acid (EDTA), 1% (w/v) polyvinylpyrrolidone (PVPP), 1 mM phenylmethylsulfonyl fluoride (PMSF) and 0.05% Triton X-100 in 50 mM potassium-phosphate buffer (pH 7.0)]. Results were expressed as enzyme units per milligram of protein.

### 2.4. Metadata Analysis of Proteomic Study in B. Cinerea Inoculated Tomatoes

In our previous study, proteome analysis took place on tomatoes exposed to ozone with or without *B. cinerea* inoculation, and the interaction of ozone and pathogen was studied with the proteome approach and described in detail previously [29]. In brief, following 1 week of storage, fruit tissue of four biological replicates for inoculated and control fruit were sampled and frozen in liquid nitrogen and stored at −80 °C before protein extraction. In wounded fruit, plant tissue was sampled from the opposite side of the wound (i.e., ‘healthy tissue’) as we targeted to study the systemic response of fruit to pathogen and not the direct response to wounding/pathogen [28]. Proteins extraction procedure has been described previously based on a phenol extraction assays [45] as modified by Tzortzakis et al. [29]. First (1-D) and second (2-D) dimensional sodium dodecyl sulfate-polyacrylamide gel electrophoresis (SDS-PAGE) analysis was carried out on 21 × 25 × 0.15 cm gels. The gels were silver stained and image analysis performed on one-dimensional SDS-PAGE gels using a Gel-Doc 1000 System (Bio-Rad Laboratories, Hercules, California, USA), enabling band quantification and image optimisation via Multi-Analyst software (Bio-Rad Laboratories, Hercules, California, USA). Once stained, 2-D gels were scanned, and images were analysed (Progenesis image analysis software, Nonlinear Dynamics, Durham, North Carolina, USA). Different gel images were compared, and the false-colour overlay function incorporated within this software was utilised. In the overlay mode, the spots of the reference gel (control: average of three) were displayed in magenta (<-1.5 fold) while the spots in the second gel (treated: average of three) were displayed in green (>1.5 fold). Overlaid matching spots appear black due to the complementary pseudocolour display, with no matching spots remaining magenta or green. Gels were cropped to remove areas of no interest, averaged in groups, the background removed, spot detection synchronised and then normalised to the average gels.

Targeted protein spots were excised from 2-D gels using an Ettan spot picker (Amersham Bioscience, Buckinghamshire, UK), subjected to mass spectrometry (MS) analysis and protein sequence determination. Peptide mass fingerprinting was conducted using a low-resolution linear MALDI-time-of-flight (MALDI-TOF) mass spectrometer (Voyager-DE^TM^ STR, Applied Biosystems, Foster City, California, USA) and identified as described previously [29]. 

### 2.5. Statistical Analysis

Data were analysed by variance (ANOVA) and before that were tested for normality. Significant differences between mean values were determined using Duncan’s multiple test (MRT) test (*p* = 0.05) following one-way ANOVA. Statistical analyses were performed using SPSS (SPSS Inc., Chicago, USA). Silver-stained spots were quantified based on their volume after gel normalisation and background subtraction, and significance differences determined using a *t*-test or non-parametric Wilcoxon test. 

## 3. Results

### 3.1. Impact of B. cinerea on Qualitative and Antioxidant Characteristics

**Figure 1:**
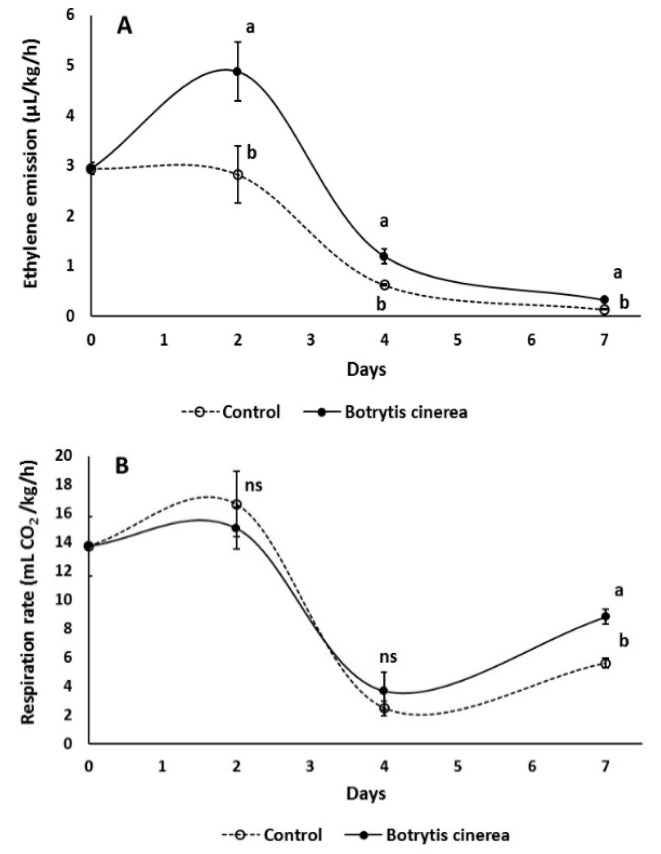
Impacts of wound-inoculation with *Botrytis cinerea* on (**A**) ethylene emission (μL/kg/h) and (**B**) respiration rate (mL CO_2_/kg/h) in tomato fruit following storage for 1 week. Fruit were maintained throughout at 11 °C and 90% relative humidity (RH). Values represent mean (± standard error) of measurements made on eight independent fruit per treatment and storage period. Symbols of *ns* indicating not significance in each day.

*Botrytis cinerea* infection significantly (*p* < 0.05) increased ethylene emission compared to the non-infected fruit (control), with greater effects during the first days after inoculation (Figure 1A), while respiration rates were increased after 1 week of storage for the *B. cinerea* infected fruit compared to the control (Figure 1B).

Quality and ripening related attributes of tomato fruit are presented in Table 1. *B. cinerea* infection softened the fruit as fruit firmness decreased in the tomato’s part neighbouring the fungi as a direct response compared with the opposite side as a systemic response to fungi as well as the control fruit. Titratable acidity decreased in SR-tomato compared with the tissue with DR and control fruit. The β-carotene and ascorbic acid content increased in DR-tomato compared with the control fruit. No differences were found in fruit weight loss (averaged in 0.29%), total soluble solids (averaged in 3.84%), ripening index (averaged in 2.15 ratio of TSS/TA), colour (averaged in values of 40.67 for L*, 24.07 for a*, 25.88 for b* and 35.38 for chroma) and lycopene (averaged in 21.47 nmol/g Fw) content among the examined treatments. The activity of FRAP increased in DR-tomato compared with the control and SR-tomato treatment. The content of total phenolics (averaged in 0.36 mg GAE/g Fw) and antioxidant activity (averaged in 0.25, and 0.09 mg Trolox/g Fw for DPPH and ABTS, respectively) did not differ among the treatments (Table 1).

**Figure 2:**
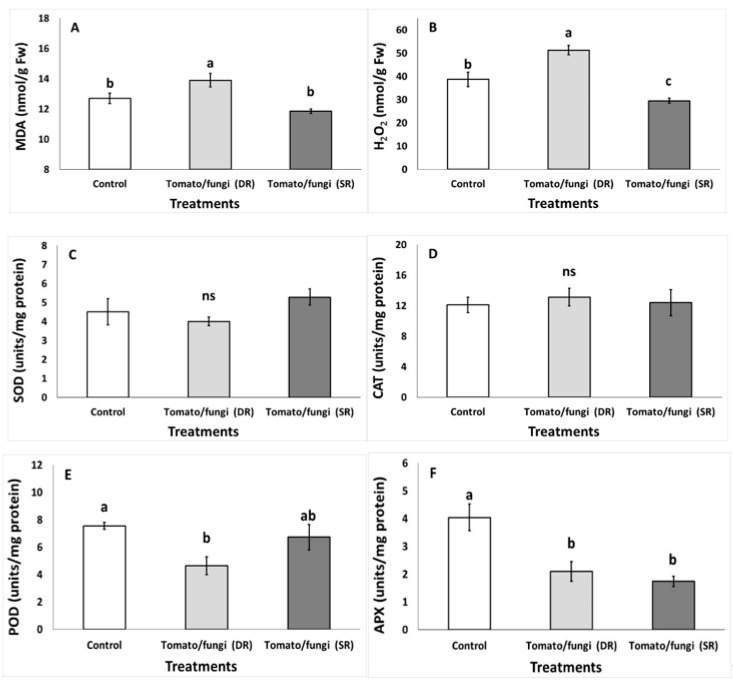
Impacts of wound-inoculation with *Botrytis cinerea* on (**A**) lipid peroxidation-MDA, (**B**) hydrogen peroxide-H_2_O_2_, (**C**) superoxide dismutase-SOD, (**D**) catalase-CAT, (**E**) peroxidase-POD and (**F**) ascorbate peroxidase-APX in tomato fruit following storage for 1 week. Fruit were maintained throughout at 11 °C and 90% relative humidity (RH). Values are mean ± standard error (*n* = 8). Mean values followed by the same letter do not differ significantly at *p* ≥ 0.05, according to Duncan’s MRT. Symbols of *ns* indicating not significance.

The lipid peroxidation, as determined with the MDA content, increased in DR-tomato compared with the control and SR-tomato treatment (Figure 2A). The H_2_O_2_ increased in DR-tomato compared with the control but decreased in SR-tomato treatment (Figure 2B). The enzyme activity for SOD and CAT did not differ among the treatments and averaged in 4.60 and 12.54 units/mg of protein (Figure 2C,D). The POD activity decreased in DR-tomato compared with the non-inoculated fruit but maintained similar levels in SR-tomatoes (Figure 2E). The APX decreased in DR- and SR-tomato compared with the non-inoculated fruit (Figure 2E). 

Total Soluble Solids (TSS); Titratable acidity (TA); Newton (N); Gallic acid equivalent (GAE); Ferric-reducing antioxidant power (FRAP); 2,2′-azino-bis(3-ethylbenzothiazoline-6-sulphonic acid (ABTS); 2,2-diphenyl-1-picrylhydrazyl (DPPH); Fresh weight (Fw).

### 3.2. Impact of B. cinerea on Protein Composition of Tomato Fruit

**Figure 3:**
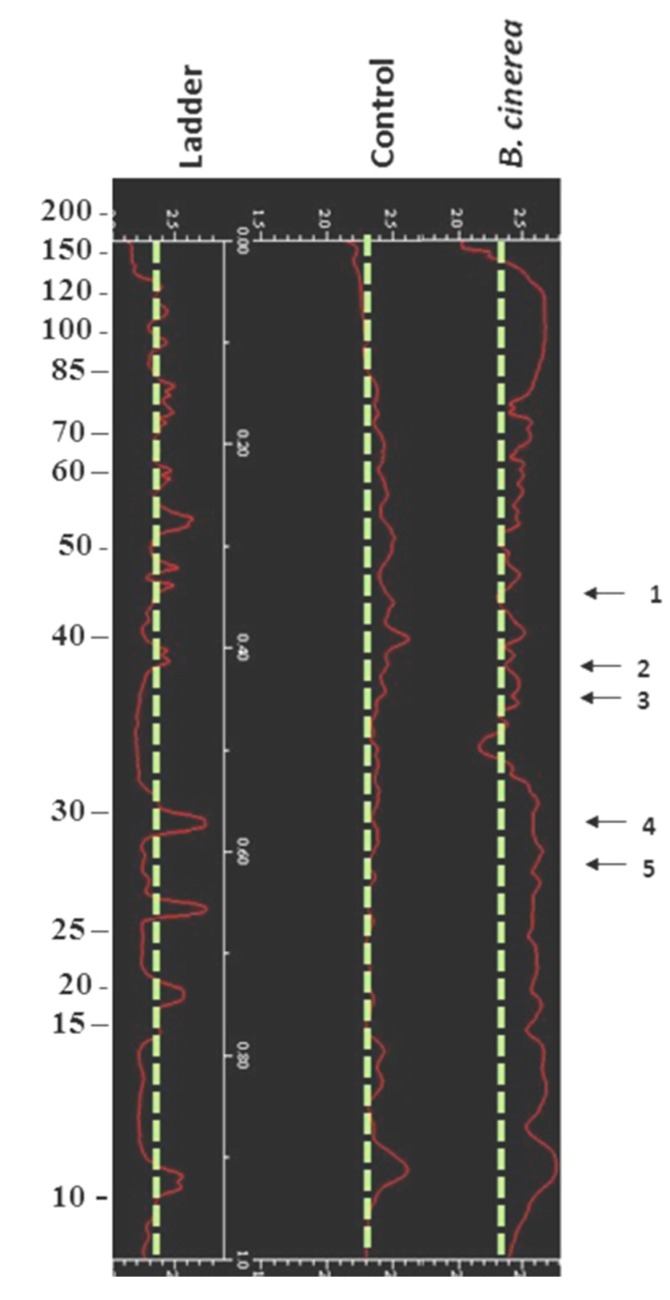
Impacts of wound-inoculation with *Botrytis cinerea* on protein content of tomato fruit. One dimensional sodium dodecyl sulfate-polyacrylamide gel electrophoresis (SDS-PAGE) gels were silver stained. Labelled arrows indicate shifts in proteins. Image analysis performed using a Gel-Doc 1000 System (Bio-Rad Laboratories, Hercules, CA, USA), enabling band quantification and image optimisation via Multi-Analyst software (Bio-Rad Laboratories, Hercules, CA, USA).

**Figure 4:**
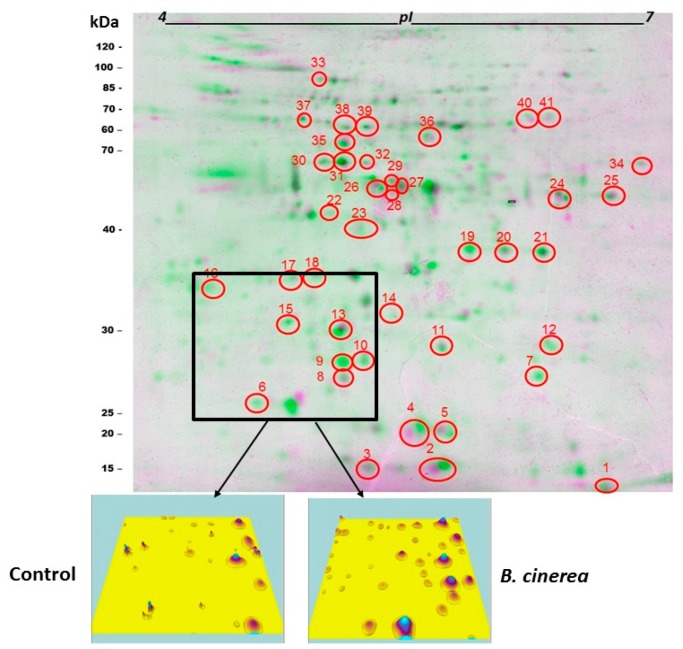
Two-dimensional gels (*p*I 4–7) illustrating differential protein composition of non-infected (control) and wound-inoculated with *Botrytis cinerea* tomato fruit. Fruit maintained 1 week at 11 °C and 90% RH. Two-Dimensional Difference Gel Electrophoresis (2D-DIGE) with controls labelled in magenta and *B. cinerea* treated labelled in green. Black spots indicate protein expression within 1.5-fold. Numbers refer to spots selected for detailed analysis.

Fungal infection significantly (*p* < 0.05) increased protein yield (up to 6.6%) compared with the control (non-infected) fruit. *B. cinerea* increased proteins levels >1.5-fold in nine proteins and decreased proteins levels >1.5-fold in 39 proteins (Table 2). This effect was detected mainly in the large proteins (>35 kDa) increase when protein profiles were developed using 1D-SDS-PAGE and visualised (Figure 3). Protein profile varied during the treatment indicating significant changes in the protein composition of the fruit subjected to *B. cinerea* infection (Figure 3). A total of 26 protein zones were resolved by 1D-SDS-PAGE. Two-dimensional gel electrophoresis revealed the resolution of up to 156 protein spots per gel, following spot filtering. Proteins were mainly visualised within the narrow 4–7 *p*I range, as identified from a 3–10 *p*I gel (Figure S2). Gels revealed a broad distribution in protein composition in wound-inoculated tomato fruit both in terms of size (10 to 110 kDaltons) and *p*I values. *B. cinerea* inoculation resulted in marked shifts in the tomato proteome following storage for 1 week compared to the relevant control (Figure 4). A three-dimension analysis on the protein volume clearly indicated the increased proteins levels in wound-inoculated tomatoes compared with the control fruit (Figure 4).

Examining the effects of *B. cinerea* on tomato proteome more precisely, 41 protein spots that had been selected for peptide fingerprinting and identified as described in our previous study [29] were used for metadata analysis. Protein expression in wound-inoculated tomatoes increased (>1.5 fold) for superoxide dismutase (SOD), chaperonin 21 (CPN21; spot 13), inorganic pyrophosphatase (PPase), malate dehydrogenase (MDH; spots 19, 20), 1-aminocyclopropane-1-carboxylate oxidase (ACO) and ascorbate peroxidase (APX1) (Table **3**). Protein expression decreased (<1.5 fold) for dihydroflavonol-4-reductase (DFR) and invertase (INV) and remained unaffected for chaperonin 21 (CPN21; spot 11), IN2-1 glutathione transferase (IN2-1), malate dehydrogenase (MDH; spot 21), glyceraldehyde 3-phosphate dehydrogenase (GAPDH), dehydrin (DHD) and hypothetical protein (HTP). 

Based on the functional association of the selected proteins, the following five metabolic groups can be identified. The first group involves constitutive proteins, such as CPN21, MDH and DHD. The second group involves proteins allied to signaling and antioxidative metabolism, such SOD, TPX, IN2-1, APX1 and ACO. The third group is associated with carbohydrate metabolism and the pentose phosphate pathway and includes PPase, INV and GAPDH. The fourth group comprises terpenoid and flavonoid-associated proteins, including FPS and DFR. The fifth group contains proteins that are associated with plant stress responses and includes ULP. One hypothetical protein (spot 28) was not grouped. Protein expression of nine of these proteins differs in wound-inoculated tomatoes with respect to the control (Table 3).

## 4. Discussion

Cantu et al. [25] reported that *B. cinerea* developed successfully in ripe (red ripe) compared to un-ripe (mature green) tomatoes, indicating that different ripening stages can alter the pathogen establishment on/in fruit. In the current study, *B. cinerea* caused marked changes in fruit quality and ripening traits, as well as changes in protein yield and composition in tomatoes. Infected tomatoes revealed increased ethylene and respiration rates compared with the non-inoculated fruit (control). A 50% increase in the gene expression related to ethylene metabolism was triggered by the *B. cinerea* in red ripe tomato fruit [23], including the *ACO* gene. However, the finding that in mature green tomatoes, *ACO* was downregulated in the *B. cinerea* contaminated fruit, can be interpreted as counteracting the plant’s efforts to control the increase in ethylene production caused by the pathogen [23]. Ripe fruit susceptibility to *B. cinerea* has been reported to be based on non-ripening (NOR) but not on ripening inhibitors (RIN) transcription factors and only partially on ethylene perception, leading to the assumption that not all pathways and events that constitute fruit ripening are susceptible to ripening [25]. Ethylene modulates plant resistance and susceptibility to pathogens. Therefore, ethylene regulates a variety of immune responses from one point of view in tandem with other signaling networks; but from another point of view, it promotes senescence or maturation, processes that encourage pathogen infection [25].

Tomato fruit ripening includes the progressive fruit softening with firmness decrease that is largely a result of the metabolism of the cell wall polysaccharides [54]. *B. cinerea* has been shown to activate fruit ripening process and softening fruit by decreasing the firmness of the commodity [23], in accordance with the decreased (up to 15.4%) firmness of infected fruit compared with the control. The level of total soluble solids did not differ in infected and control fruit, possibly due to the red-ripe stage of the fruit and/or the short period of 1 week. However, carbohydrate breakdown takes place during fruit ripening [7]. Bui et al. [55] reported that vitamin C content maintained at 5 days and decreased at 14 days on the infected with *B. cinerea* apple tissue. Similarly, in the present study, ascorbic acid on infected tissue (DR) maintained similar levels with the control after 7 days of storage but decreased in the tissue opposed to infection (SR). FRAP antioxidant activity increased in DR-tomato compared with the control and SR-tomato treatment, but total phenols, DPPH, ABTS remained unaffected among treatments. Indeed, Bui et al. [55] reported that total phenolics decreased in the *B. cinerea* infected apple tissue, and these differences can be attributed to the longer storage period and/or different commodities.

Differentially expressed genes revealed functions during tomato inoculations that are likely to be pathogenic, such as catabolisms of ROS (e.g., SOD, CAT, POD) and cell wall breakdown molecules, including cellobiose and lignin [56]. ROS are rapidly produced by the host after pathogen detection, activating downstream signaling of different defense responses [57]. However, by overwhelming the host with their own ROS production, necrotrophic pathogens can exploit this ROS response [58], underlining the complexity of the plant–pathogen interaction system. In addition to ROS generation machinery, fungal pathogens must be shielded from the infection site’s oxidative stress, with activation of enzymatic and non-enzymatic mechanisms [59]. SODs activation is the first step to convert O_2_^−^ into H_2_O_2_, and then H_2_O_2_ can be converted to water by either CATs or peroxidases (GPXs or PRXs), and *B. cinerea* revealed upregulation of mechanisms for the H_2_O_2_ catabolism [56], evidence that was also obtained in the present study.

There is histochemical evidence during pathogenesis that *B. cinerea* produces O_2_^−^ and H_2_O_2_ in hyphal germ tubes at the site of the infection, and that its superoxide dismutases may be involved in pathogenicity [59]. In *B. cinerea*, SOD- and CAT-related genes were upregulated, scavenging the hydrogen peroxide production [56] in wound-inoculated tomatoes after 1 and 3 days post-inoculation, and this is confirming the present outcomes, as SOD-related protein was increased in wound-inoculated tomatoes after 1 week of storage. 

Hydrogen peroxide levels increased in DR-tomato compared with the control and SR-tomato treatment, indicating oxidative stress through the increased MDA levels. The H_2_O_2_ had been partly detoxified at earlier stages, possibly with the early activation of SOD initially and then by CAT, as both enzymes remained at similar levels after 1 week of storage, while other involved enzymes (POD, APX) were changed. Bui et al. [55] reported that SOD increased in the *B. cinerea* infected apple tissue after 14 days, but CAT did not differ in the *B. cinerea* infected apple tissue and the control, in accordance with the present findings as CAT remained at similar levels for the healthy and DR- and SR-tomato treatments. The levels of APX and POD were decreased (consumed), possibly due to the increased oxidative stress as indicated by the revealed increased MDA and H_2_O_2_ levels in DR-tomato after 1 week of storage on infected fruit. In addition to the enzymatic antioxidant mechanisms, obviously activated as above described, the tomato fruit also brought about the non-enzymatic antioxidant mechanism to trigger ROS, with increased levels of ascorbic acid and *β*-carotene. 

Fungal infection with *B. cinerea* revealed stress conditions and resulted in a 6.6% increase in protein yield, and the downregulation of 39 proteins, compared with the control treatment. Fifteen proteins of known function were tentatively identified, as described in Tzortzakis et al. [29]. Interestingly, signaling proteins (ACO) related to the ethylene production, a well-known ripening hormone and antioxidant proteins (APX, SOD) which are involved in the detoxification of ROS, accelerated with the *B. cinerea* infection, indicating an enhanced ripening process and stress environment. Ascorbate peroxidase is induced after exposure to both abiotic (radiation, drought, wounding, extreme temperature) and biotic (pathogen attack) stress conditions [29,60,61,62]. However, *B. cinerea* infected tomatoes increased gene expression of *ACO1* [11], but when the infected tomatoes were exposed to ozone, they revealed decreased ACO levels, as ozone retarded ethylene biosynthesis/action in both proteins and gene levels [11,29]. However, TPX and IN2-1 being oxy-responsive proteins did not appear sensitive to wound-inoculation with *B. cinerea,* but induced in ozone-exposed tomatoes [29]. IN2-1 protein was induced in tomato fruit in response to blossom-end-rot [46] but remained unaffected in herbicide treated maize (*Zea mays* L.) and soybean (*Glycine max* L. Merr.) [63].

Inorganic pyrophosphatase, involved in carbohydrate metabolism, increased in the wound-inoculated tomatoes. Invertases are a group of related enzymes that hydrolyze sucrose to hexose sugars with a contribution to tomato fruit quality, in terms of both flavour and acids [47]. INV decreased in wound-inoculated tomatoes, and possibly producing sweeter tomatoes, in accordance with previous findings with heat stress on tomatoes. Low levels of acid invertase are associated with low levels of hexose and high levels of sucrose in tomatoes [47]. However, ozone-treated tomatoes had almost the same levels on PPase and INV even though ozonated tomatoes were sweeter [7,29]. 

Constitutively-expressed proteins tended to be either increased (CPN21 and MDH) or remained unaffected (DHD) by wound-inoculation. CPN function is related to the optimal folding and protection of proteins under normal and stress conditions [64]. DHD is scavenging hydroxyl radicals [65]. However, it was unaffected in the present study, possibly replaced by the induction of other antioxidant scavengers, such as APX and SOD. The expression of proteins involved in terpenoid or flavonoid biosynthesis differed in infected tomatoes and control, with a decrease of dehydroflavonol-4-reductase, which catalyzes the last step in the flavonoid-biosynthesis pathway leading to anthocyanins and proanthocyanidins [66].

Several studies have been carried out on the molecular genetics underlying bacterial– and fungal–plant pathogenesis [11,67], but few studies have discussed proteome modifications associated with such interactions [29,36,68]. Currently, proteomic analysis has been widely used to investigate the resistance mechanism of fruit induced by various exogenous factors in response to fungal pathogens, such as peach induced by salicylic acid and yeast [69] and sweet cherry induced by salicylic acid [70]. Several studies report differential protein expression in susceptible host plants [71,72], with one particular plant pathogen *Xylella fastidiosa* research revealing significant changes in cellular and extracellular bacterial proteins, including toxins, adhesion-associated proteins, antioxidant enzymes and proteases [73]. 

## 5. Conclusions

In the present work, the proteome analysis of the tomato fruit proteins has been carried out together with all metabolites involved in systemic response and the antioxidative mechanism of the fruit tissue with increased SOD and APX proteins levels. Fungal infection accelerated the ripening process of the fruit with increased ethylene and respiration rates. Metabolic changes in fruit quality were more pronounced in the infected part of the fruit as a direct response to the pathogen compared to the systemic response, with a decrease in firmness and increase in lipid peroxidation and hydrogen peroxide and induced fruit antioxidative metabolism (i.e., β-carotene, ascorbic acid) to trigger the pathogen stress. Our study highlights the importance of quality responses (direct versus systemic) of fruit under pathogen infection and contributes to understanding the plant–pathogenic fungus proteome under systemic response.

## Figures and Tables

**Table 1:** Impacts of wound-inoculation with *Botrytis cinerea* on quality and ripening related attributes of tomato fruit stored for 1 week at 11 °C/90% relative humidity (RH). In wounded fruit, plant tissue was measured/sampled from the infected side of the fruit (direct response-DR) and from the opposite side of the wound (systemic response-SR) of fruit. Mean values (± standard error) followed by the same letter in each row do not differ significantly, according to Duncan’s multiple test (MRT).

Quality Parameters	Control	Tomato Tissue with Fungal Lesion (DR)	Tomato Tissue Without Fungal Lesion (SR)
Weight loss (%)	0.335 ± 0.030 a	0.259 ± 0.031 a	
Fruit firmness (N)	11.08 ± 0.43 a	9.37 ± 0.46 b	10.99 ± 0.55 a
Total soluble solids (%)	3.80 ± 0.20 a	3.72 ± 0.18 a	4.01 ± 0.02 a
Titratable acidity (citric acid %)	1.76 ± 0.06 b	1.60 ± 0.08 b	2.01 ± 0.06 a
Ripening index (TSS/TA)	2.15 ± 0.07 a	2.34 ± 0.19 a	1.98 ± 0.06 a
Colour L *	40.98 ± 0.37 a	40.48 ± 0.61 a	40.56 ± 0.59 a
Colour a *	23.44 ± 0.61 a	24.63 ± 0.82 a	24.15 ± 1.08 a
Colour b *	25.60 ± 0.71 a	26.73 ± 0.68 a	25.32 ± 0.44 a
Chroma	34.74 ± 0.73 a	36.37 ± 0.95 a	35.03 ± 0.99 a
Ascorbic acid (mg/g Fw)	17.66 ± 1.16 b	21.93 ± 1.77 a	14.15 ± 1.00 b
Lycopene (nmol/g Fw)	17.14 ± 2.69 a	27.34 ± 3.80 a	19.93 ± 2.95 a
β-carotene (nmol/g Fw)	6.74 ± 1.02 b	10.66 ± 0.49 a	7.75 ± 0.93 ab
Phenols (mg GAE/g Fw)	0.39 ± 0.031 a	0.36 ± 0.043 a	0.34 ± 0.019 a
FRAP (mg Trolox/g Fw)	3.44 ± 0.12 b	4.38 ± 0.31 a	3.31 ± 0.20 b
DPPH (mg Trolox/g Fw)	0.22 ± 0.00 a	0.30 ± 0.04 a	0.24 ± 0.02 a
ABTS (mg Trolox/g Fw)	0.07 ± 0.01 a	0.11 ± 0.01 a	0.11 ± 0.00 a

Total Soluble Solids (TSS); Titratable acidity (TA); Newton (N); Gallic acid equivalent (GAE); Ferric-reducing antioxidant power (FRAP); 2,2′-azino-bis(3-ethylbenzothiazoline-6-sulphonic acid (ABTS); 2,2-diphenyl-1-picrylhydrazyl (DPPH); Fresh weight (Fw).

**Table 2:** Impacts of *Botrytis cinerea* on protein yield and protein levels of tomato fruit stored for 1 week at 11 °C/90% relative humidity (RH). Mean values (± SE) followed by the same latter in each row do not differ significantly, according to Duncan’s multiple test (MRT). Values in parenthesis represent shifts (compared to control) of individual proteins by at least 1.5 fold.

Proteins Level	Control	*B. cinerea* infected Tomatoes
Protein yield (μg/g Fw)	140.8 ± 5.31 b	150.1 ± 4.98 a
Increased proteins by fungi		29 ± 5 (9)
Decreased proteins by fungi		60 ± 4 (39)
Novel by fungi		0 ± 0 (0)
Total		89 ± 2 (48)

**Table 3:** Putative identification of proteins responsive to wound-inoculation with *B. cinerea.* Protein expression (**↓**) downregulated (<-1.5-fold); (**↑**) upregulated (>1.5-fold) and (-) in unchanged behaviour within 1.5-fold. Numbering refers to spots selected for detailed analysis. Protein analysis failed for spots 6–10, 12, 16, 18, 22–24, 26–27, 30, 33–34, 36–41.

Spot No	Identification, Putative Function, Species/EC ^b)^	Protein Expression
1	Superoxide dismutase (Cu-Zn), chloroplast precussor, ***Lycopersicon esculentum***/1.15.1.1	**↑**
2	Farnesyl pyrophosphate synthase, synthesis of farnesyl pyrophosphate, ***L. esculentum***/2.5.1.1	-
3	Ulp1 protease-like, ***Oryza sativa*** (japonica cultivar-group)/	-
4	Dehydrin 2, ***Pisum sativum***	-
5	Thioredoxin peroxidase, ***L. esculentum***/1.11.1.-	-
11	Chaperonin 21 precursor, ***L. esculentum***	**-**
13	Chaperonin 21 precursor, ***L. esculentum***	**↑**
15	IN2-1 glutathione transferase, ***Arabidopsis thalian**a*/2.5.1.18	**-**
17	Inorganic pyrophosphatase, ***A. thaliana***/3.6.1.1	**↑**
19	Putative NAD-dependent malate dehydrogenase, ***Solanum tuberosum***/1.1.1.37	**↑**
20	Putative NAD-dependent malate dehydrogenase, ***S. tuberosum***/1.1.1.37	**↑**
21	Putative NAD-dependent malate dehydrogenase, ***S. tuberosum***/1.1.1.37	**-**
25	Glyceraldehyde 3-phosphate dehydrogenase, ***L. esculentum***/1.2.1.12	**-**
28	Hypothetical protein, ***O. sativa*** japonica cultivar-group)/	**-**
29	1-Aminocyclopropane-1-carboxylate oxidase homolog (protein E8), ***L. esculentum***/1.14.17.4	**↑**
31	L-ascorbate peroxidase 1 cytosolic, ***A. thaliana***	**↑**
32	dihydroflavonol-4-reductase, ***L. esculentum***/1.1.1.219	**↓**
35	Invertase, ***L. esculentum/***3.2.1.26	**↓**

## Supplementary Materials

The following are available online at https://www.mdpi.com/2076-2607/7/12/681/s1, Figure S1: Healthy and *Botrytis cinerea* infected tomato fruit after 1 week of storage at 11 °C and 90% relative humidity. Figure S2: Two-dimensional gels (*p*I 3-10) illustrating the protein composition of tomato fruit. Fruits maintained 1 week at 11 °C and 90% RH.
