# Supplementary material for: Physiological and Proteomic Approaches to Address the Active Role of Botrytis cinerea Inoculation in Tomato Postharvest Ripening
Source: Microorganisms. 2019 Dec 11;7(12):681. doi: 10.3390/microorganisms7120681 (PMC6955909; doi:10.3390/microorganisms7120681)
Supplement: Supplementary file 1 [file microorganisms-07-00681-s001.pdf]

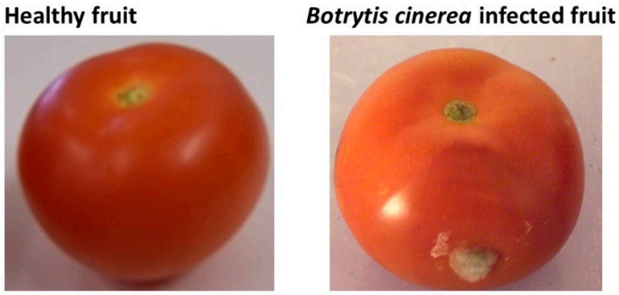

Figure S1. Healthy and Botrytis cinerea infected tomato fruit after 1 week of storage at 11 ºC and 90% relative humidity.

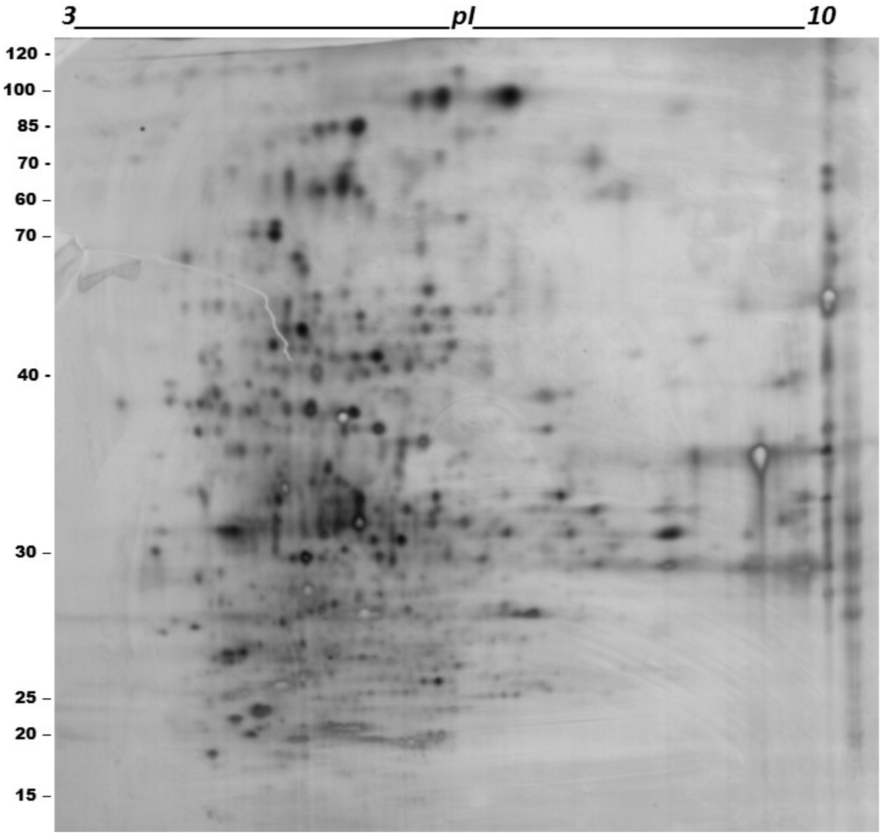

Figure S2. Two-dimensional gels (*p*I 3-10) illustrating the protein composition of tomato fruit. Fruits maintained 1 week at 11 ºC and 90% RH.
